# Treatment with Soteria-elements in acute psychiatry—Effectiveness for acutely ill and voluntarily treated patients

**DOI:** 10.3389/fpubh.2023.1118522

**Published:** 2023-02-13

**Authors:** Philine Fabel, Theresa Wolf, Helena Zyber, Julian Rubel, Maria C. Jockers-Scherübl

**Affiliations:** ^1^Department of Psychiatry and Psychotherapy, Academic Teaching Hospital of the Charité Berlin, Oberhavel Kliniken GmbH, Hennigsdorf, Germany; ^2^Department of Psychology and Sports Sciences, Justus-Liebig-University Giessen, Giessen, Germany

**Keywords:** Soteria, acute psychiatry, schizophrenia, inpatient treatment, medication dosage

## Abstract

**Objective:**

This article aims at evaluating the treatment outcomes of acute psychiatric patients before and after the implementation of Soteria-elements in an acute psychiatric ward. The implementation process resulted in an interconnected small locked and much larger open area, enabling continuous milieu therapeutic treatment by the same staff in both areas. This approach enabled the comparison of structural and conceptual reconstruction regarding treatment outcomes of all voluntarily treated acutely ill patients before (2016) and after (2019). A subgroup analysis focused on patients suffering from schizophrenia.

**Methods:**

Using a pre-post design, the following parameters were examined: total treatment time, time in locked ward, time in open ward, antipsychotic discharge medication, re-admissions, discharge circumstances, and treatment continuation in day care clinic.

**Results:**

Compared to 2016, there was no significant difference in the total time of stay in the hospital. However, data show a significant decrease of days spent in locked ward, a significant increase of days in open ward, a significant increase of treatment discontinuation but without an increase of re-admissions, and a significant interaction of diagnosis and year regarding the medication dosage, resulting altogether in a reduction of antipsychotic medication for patients suffering from schizophrenia spectrum disorder.

**Conclusion:**

The implementation of Soteria-elements in an acute ward facilitates less potentially harmful treatments of psychotic patients, likewise enabling lower dosages of medication.

## 1. Introduction

Based in the movement of anti-psychiatry in the 1960's, Lauren Mosher provided an alternative to the traditional psychiatric treatment of patients suffering from schizophrenia by establishing the first Soteria House in the 1970's in San Francisco. Mosher and colleagues aimed to implement a treatment, which instead of the traditional medical understanding of illness and treatment was based on a psychosocial approach (1, 2). This included not only an abandonment of the complex wards and authoritarian social structures often found in traditional psychiatry at that time. Mosher and colleagues created a space for six selected patients (first psychotic episode, age between 14–30 years), who were treated in a community house with their own room, high-frequent care by medical laymen and without any antipsychotic medication (2). Luc Ciompi adapted the idea and founded the Soteria Bern in 1984. Since Ciompi postulated stress as an important factor causing psychotic episodes in his theory of affect logic (3), all treatment interventions aimed at a maximum reduction of stress and were supposed to take place in a normal, non-psychiatric setting (4). Thus, he created eight treatment principles, that required (1) a small, stress reducing and transparent milieu, (2) high-frequency care during the psychotic episode (“being with”), (3) conceptual and personal continuation during the treatment, (5) close cooperation with family and relatives, (5) transparent communication between the patient, family and staff regarding the disease, treatment, risks and chances, (6) elaboration of realistic common goals and perspective with patient and relatives, (7) the least possible dosage of antipsychotic medication, with the goal of the patient's controlled self-medication and (8) outpatient after care and relapse prevention for at least 2 years (4). The original concept of Soteria treatment was specifically designed for patients suffering from psychotic disorders.

The empirical evidence of the effectiveness of Soteria-treatment regarding equivalent or better outcomes of patients is still poor (4). Bola and Mosher conducted the most detailed analysis in 2003 (1, 5), showing in a randomized controlled trial (RCT), that Soteria-patients showed equivalent or better outcomes after a 6-week-treatment without medication, compared to patients treated as usual. Even within a 2-year follow-up period, there were small to medium effects in the general functioning level (1). Ciompi et al. (6) showed similar outcomes in Soteria-patients with no or low dosage of antipsychotic medication compared to patients treated as usual in a 2-year follow-up period, even though this finding is based on a small sample size of index-patients (*n* = 14). Further research is based on qualitative analysis (4, 7, 8), getting to similar results of comparable outcomes between Soteria- and standard-care patients. There appears to be an agreement about the need for more empirical analysis of the effectiveness of Soteria-treatment as an alternative to standard care.

To evaluate the impact of the Soteria-concept, its limits have to be taken into account. In the traditional concept, the treatment was only offered to selected patients, also not all of them could be held in the Soteria (since the foundation of Soteria Bern, 10–15% of a total of around 2,000 treated patients had to be transferred to regular care because of reduced controllability; 3, 4). In the 1990's, first attempts were made to establish the Soteria idea in acute regular care. The Westfälische Klinik Gütersloh was the first hospital trying to integrate the principles of Soteria-treatment in acute regular care by implementing both structural (i.e., ward with open door, combined living room and kitchen, soft room, continuous treatment staff) and conceptual (i.e., negotiating instead of treating, low dosage application of antipsychotics, “being with,” abandonment of coercive measures) changes. In qualitative accompanying research, a change in the ward atmosphere, growing acceptance by patients and relatives as well as a notable reduction of coercive measures [10% compared to other wards; (2, 9)] were observed. Unfortunately, there are no empirical data available and due to changes in administration, the concept in Gütersloh could not be continued.

There are numerous initiatives to offer Soteria-treatment to a larger number of patients in Europe, where the patients are selected (e.g., Soteria Bern, Soteria Berlin, Soteria Klinikum München-Ost, Soteria im Zentrum für Psychiatrie Reichenau, Soteria an der Münsterklinik Zwiefalten, also see https://Soteria-netzwerk.de/Soteria-einrichtungen). To our knowledge, the acute psychiatric ward with Soteria-elements in Hennigsdorf (Oberhavel Kliniken) is the only one in Europe in acute regular care. It is also the only ward for acutely ill psychiatric patients in the Hennigsdorf hospital (other than the geropsychiatric ward). A transfer to other wards in case of reduced controllability is thus not possible. Previously published data (10) demonstrated a significant benefit for legally accommodated patients treated with Soteria-elements in acute care in the Oberhavel Klinik Hennigsdorf. The present article however, focusses on the effect of Soteria-elements in acute care on all patients in the same acute ward, who were treated voluntarily and who constitute the vast majority. Further subgroup analysis focusses on all the schizophrenic patients who were treated in our hospital in 2016 and 2019 on a voluntary basis, thereby comparing the treatment outcomes before and after the implementation of Soteria-elements.

## 2. Methods

### 2.1. Implementation of Soteria-elements in acute care in the Oberhavel Klinik Hennigsdorf

In 2017, the acute ward of the hospital with 24 beds and optionally closed door was spatially and conceptually restructured into a ward with Soteria-elements with the aim to offer a disorder specific treatment for psychotic patients on the acute psychiatric ward. Soteria is Greek for salvation, safety, deliverance. Soteria treatment in acute care is supposed to be carried out in a small, stress reducing milieu that promotes interpersonal contacts and enables an individual companionship during the psychotic episode [“being with”; (11)]. To implement the Soteria-elements, major spatial and conceptual changes were made. After the re-opening in 2018, the acute psychiatric ward with Soteria-elements comprises a larger open area with 15 beds and a small protected area with 6 beds. Since the two areas are interconnected, it is possible for the patients to switch between those two areas according to their individual needs (i.e., as soon as someone was able to keep to agreed conditions, a transfer to the open area of the ward took place), allowing treatment continuation by the same members of the therapeutic team. A return to the open ward can take place gradually (e.g., temporarily spending the nights in the protected area and still being part of the larger patients' community in the open area).

The fundamental conceptual changes made were based on the criteria for “ward with Soteria-elements” of the Soteria Fidelity Scale (12). Major changes include the establishment of milieu therapy in everyday treatment. This required a development of the staff's attitude toward the patients in establishing a recovery-oriented mindset, which implies an accepting, supportive and less hierarchical mind-set toward the patients. High frequent de-escalation trainings were conducted. Antipsychotic drug treatment is discussed and agreed upon in an open dialogue with the patients. The staff is meant to support and accompany the patient throughout the psychotic episode and to help find a meaning in the individual experience. A crucial element of Soteria-treamtent is “being with” – the continuous companionship during the acute psychotic episode. Therefore, substantially/up to 4 times more group therapy was implemented into the schedule to guarantee more than 50% of working hours directly with the patients. Besides disorder specific group therapies there are numerous occupational therapies in the open and protected area of the ward to train everyday skills and improve cognitive abilities and social skills. A large dining area for all patients enables interpersonal encounters and milieu therapeutical offers. Daily breakfast, lunch and dinner is planned, prepared and consumed by the patients and staff together. Since there are still other acute patients on the acute psychiatric ward, time with the patients must be planned—in contrast to traditional Soteria houses. Therefore, daily group therapies are defined in the therapy plan. Patient participation is always agreed on individually according to the current abilities and needs. In the same manner, a voluntary shift between the open and protected area is discussed with the patient and the team.

Multi-professional working group meetings monitoring the process took place weekly. Frequent internal and external trainings as well as external supervision were provided. There was no change in the ward's and hospital's senior staff. Professional exchange with colleagues from the above-mentioned Soteria facilities supported the process.

Relapse prevention is given by the admission to the hospital's psychiatric outpatient clinic and close cooperation with local social organizations. The structures of our other inpatient wards and day-care clinics remained essentially the same. However, in the same year, an additional ward for psychotherapeutic crisis intervention was established in the hospital, focusing on short term interventions, predominantly for patients with borderline personality disorders and PTSD and the like.

The Hennigsdorf Hospital is part of the Oberhavel Hospitals. The Department of Psychiatry and Psychotherapy offers a total of 101 beds and 57 day-care clinic places at the locations Hennigsdorf, Oranienburg and Gransee as well as a large outpatient clinic. The department is responsible for the psychiatric treatment in the Oberhavel catchment area, which is located in the federal land of Brandenburg in the North of Berlin and has a population of about 202,000. The treatment offered comprises a disorder-specific group therapy concept and, additionally to the acute ward with Soteria-elements and the abovementioned ward for crisis intervention, there is an interdisciplinary geropsychiatric ward, a ward specifically treating affective disorders, and a ward for addiction and comorbid disorders. This conceptual re-organization allows a treatment with focus on the specific disorders. Thus, patients with an acute psychotic disorder (legally accommodated patients according to state law or legal guardian law as well as help-seeking patients on a voluntary basis) in the Oberhavel catchment area can thus be treated in a small sized acute ward with Soteria-elements. Since it is the only acute psychiatric ward of the catchment area, patients with other severe mental health crises are admitted, too, following the platform model (13). Nevertheless, the aim of the reconstruction was the specification of treatment interventions for a relatively homogenous group of patients with psychotic disorders. This complies with the concept of Mosher, who developed the Soteria-treatment specifically for psychotic patients. The Soteria Fidelity Scale (12) demands a majority of psychotic patients for wards with Soteria-elements.

### 2.2. Evaluation of the implementation

In June 2018 the acute care ward with Soteria-elements was opened after the implementation and was officially recognized as a “ward with Soteria-elements.” The acknowledgment took place by the evaluations using the Soteria Fidelity Scale (12) comprising the dimensions “spatial setting,” “care team,” “treatment setting” and “Soteria everyday life.” In addition, the International Working Group Soteria (IAS), including professor Luc Ciompi, came for an audit to our hospital to evaluate the implementation. This resulted in their classification as recognized an acute psychiatric ward with Soteria-elements (also see https://soteria-netzwerk.de/soteria-einrichtungen).

The effect of the implementation of Soteria-elements in the acute ward in Hennigsdorf Oberhavel Kliniken on the treatment outcomes was evaluated regarding the total treatment duration, the treatment time in the protected and open areas of the ward, the medication dosage, the number of stays per year (“revolving door effect”), the discharge circumstances, and the transfer to day-care clinic. The object of this study is the evaluation of implementation of Soteria-elements in an acute psychiatric ward mandated to provide regional healthcare service. Additionally, the aim is to provide new insights into the effectiveness of Soteria-treatment for patients suffering from schizophrenia and psychotic disorders. Thus, the following analyses refer to all the voluntarily treated patients overall, as well as to the relevant subgroup.

### 2.3. Data analysis

Data were gathered *via* the hospital's internal information system and extracted from the discharge letters, complemented by the daily documentation records. When admitted to hospital voluntarily patients sign a treatment contract containing the approval of the retrospective evaluation of clinical outcome in a pseudonymised way. Collected data of all the patients admitted to the acute psychiatric ward between 1st of January and 31st of December in 2016 (t0, before the reconstruction) and 2019, (t1, after the reconstruction) respectively, have been analyzed in a pre-post design. The following dependent variables were examined: duration of total stay, duration of voluntary stay in the protected area, duration of stay in the open area, neuroleptic dosage measured *via* chlorpromazine equivalents (CPZE, based on Benkert and Hippius, 14), number of stays per year (“revolving door effect”), discharge circumstances, and transfer to day-care clinic. The data processing was carried out anonymously.

Data gathering was run with Microsoft Excel and the statistical analysis with IBM SPSS 22.0. The research focused on group differences between t0 (2016) and t1 (2019). Therefore, uni- or multivariance analysis of variances (ANOVA or MANOVA) were run for metric dependent variables, Bonferroni adjusted for multiple testing. Since none of the dependent variables were normally distributed and there were several outliers and multivariate outliers, Kruskal-Wallis test was used for non-parametric testing. To evaluate the effect of implementation of Soteria-elements in the acute ward, those patients with a total stay-duration of <24 h (mostly intoxicated patients admitted for one night) were excluded from the analysis as it must be assumed that those patients could not have benefitted from the therapeutical concept. Other outliers regarding treatment time or medication dosage were not excluded from the analysis since the data represent the realistic care situation in an acute psychiatric ward, where the treatment with Soteria-elements is supposed to apply. Excluding these elements would diminish the external validity. Even though ANOVA is shown to be robust against the violation of normally distributed data (14–16), Kruskal-Wallis tests were used to test for effect consistency. Since non-parametric testing showed robust directions of all the effects, results of parametric analysis are reported. Differences in categorical variables were tested *via* chi^2^ tests. To keep the analysis straightforward we analyzed the outcomes of the patient's first stay per year, tackling multiple stays in separate variables (number of stays per year, re-admission rate).

The number of patients with schizophrenia spectrum disorder who were treated voluntarily in both years on the acute psychiatric ward was considerably small (all diagnoses: *n* = 34, patients with schizophrenia: *n* = 7). Because of the limited validity and interpretability of statistical comparisons, those patients were excluded from this article.

All diagnoses were made according to ICD-10 (17) and DSM-5 (18) criteria by trained psychiatrists. All the patients who were treated on the acute psychiatric ward needed intensive treatment in all three dimensions following the platform model (13).

CPZE values (19) were determined for all prescribed oral and depot antipsychotic medication. Individual CPZE values per patient were thus generated to enable a comparison between the years 2016 and 2019.

The circumstances of discharge were coded as follows: 1 = planned discharge, 2 = discharge upon patient's own request, 3 = discharge against medical advice, 4 = premature termination by patient, 5 = transfer to other ward, 6 = no further treatment offer.

To analyze whether the circumstances of discharge changed depending on the treatment, three categories were created: “by agreement” (condition 1, 2, 5 = we did not see further treatment on our ward as necessary), “discontinuation” (4, 6 = attrition), and “against medical advice” (3).

In 2019, all patients diagnosed with schizophrenia spectrum disorder were admitted to our acute psychiatric ward with Soteria-elements. Considering that in 2016, there was no treatment offer in an open area on the acute psychiatric ward, an assessment of a change in treatment time in the open sector was not possible (days in open ward = 0). To address this matter, an additional *post-hoc* data collection was run, filtering all patients with a main diagnosis of schizophrenia, who in 2016 were initially admitted to other open wards, either because of a lower level of severeness or because of the wards' capacities. 28 patients were thus included. By including those patients into the main analysis of 2016 in addition to those admitted initially to the protected ward and further being transferred to open wards, a comparison of treatment time in the open ward without Soteria-elements (in 2016) and with Soteria-elements (in 2019) is possible.^1^ In 2019, the staff of the ward with Soteria-elements accompanied (through frequent consultation of the hospital's internal ethic committee) a long-term patient on his way to death, who suffered from severe somatic illness rejecting medical treatment because of manifested psychotic delusions. This patient was excluded from the analysis.

This article has two objectives: the evaluation of the implementation of Soteria-elements in a hospital's only acute ward as well as new findings specifically regarding the efficiency of the treatment with Soteria-elements of patients suffering from schizophrenia. Therefore, analyses aim at different groups of patients: (1) all patients treated on the acute ward with Soteria-elements, regardless of their diagnoses—admitted due to severity of illness, (2) all patients suffering from schizophrenia spectrum disorder at whom the treatment concept of the acute ward with Soteria-elements originally aims at.

## 3. Results

### 3.1. Description of the sample

In 2016, *n* = 341 patients and in 2019 *n* = 173 patients were included in the main analysis. Table 1 shows the sociodemographic data as well as the distribution of diagnoses per year of all patients. In 2019, the patients treated on the acute psychiatric ward—regardless of their diagnosis-were significantly younger [*F* (1,512) = 23.539, *p* < 0.001]. This effect is consistent for the subgroup of patients diagnosed with schizophrenia spectrum disorder treated in 2019 [*F*(1,115) = 10.213, *p* = 0.002]. Considering all patients except those with schizophrenia, this effect remained the same [*F* (1,395) = 13.186, *p* < 0.001]. Thus, age was taken as covariate in every calculation.

**Table 1:** Sample characteristics.

	**2016**	**2019**	**Statistics**
Sample size (*n*)	341	173	
Age *M* (SD)	52.12 (18.06)	44,14 (16.71)	*F* (1,512) = 23.539, *p* < 0.001^***^
Gender in % (m/f)	58.7/41.3	63.0/37.0	*χ^2^*(1) = 0.908, *p* = 0.341
**Diagnosis** ***n*** **(%)**			*χ^2^*(8) = 28.279, *p* < 0.001^***^
Organic mental disorders	72 (21.1%)	14 (8.1%)	
Substance use disorders	84 (24.6%)	31 (17.9%)	
Schizophrenia	66 (19.4%)	51 (29.5%)	
Depression	29 (8.5%)	19 (11.0%)	
Trauma, stress disorders, anxiety disorders	5 (1.5%)	3 (1.7%)	
Personality disorders + additional disorder	15 (4.4%)	7 (4.0%)	
Psychotic disorder + comorbid SUD	35 (10.3%)	34 (19.7%)	
Depression + comorbid SUD	25 (7.3%)	9 (5.2%)	
Manic episode	10 (2.9%)	5 (2.9%)	

*n*, Number of subjects; M, mean value; SD, standard deviation;

*** *p* < 0.001.

There was no significant difference regarding the gender of all patients [χ^2^(1) = 0.908, *p* = 0.341] (see Table 1). Also for the subgroup of schizophrenic patients, the distribution of gender did not differ significantly [χ^2^(1) = 2.550, *p* = 0.110]. Sociodemographic data of the subgroup are presented in Table 2.

**Table 2:** Sample characteristics of patients with schizophrenia spectrum disorder.

	**2016**	**2019**	**Statistics**
Sample size (*n*)	66	51	
Age *M* (SD)	49.27 (14.19)	40.86 (14.01)	*F* (1,115) = 10.213, *p* = 0.002^**^
Gender in % (m/f)	43.9/56.1	58.8/41.2	*χ^2^*(1) = 2.550, *p* = 0.110

*n*, Number of subjects; *M*, mean value; SD, standard deviation;

** *p* < 0.01.

The distribution of diagnoses did differ significantly between the years [χ^2^(8) = 28.279, *p* < 0.001] (see Table 1).

In the following, the results of the between subject-design analysis will be reported.

### 3.2. Between subject design

A one-way MANOVA showed a statistically significant difference between the years on the combined dependent variables [*F* (5,504) = 17.429, *p* < 0.001, *partial* η^2^ = 0.147, *Wilk's* Λ = 0.853]. Follow up ANOVAs were run.

There was a significant difference between 2016 and 2019 regarding the frequency of treated diagnoses [χ^2^(8) = 28.279, *p* < 0.001] on the acute ward, resulting in an increase of patients suffering from schizophrenia and psychotic disorders with substance use disorders (SUD) and a decrease of patients primarily with organic mental disorders and SUD as main diagnosis (see Table 1). Due to the implementation of disorder specific treatment offers on the other wards (i.e., ward for short-term crisis intervention and the geropsychiatric ward) it was possible in the first place, to offer a psychosis specific treatment for those patients in need in 2019.

#### 3.2.1. Total duration of stay

The duration of voluntary treatment of all patients admitted to the acute psychiatric ward did not differ significantly between the years [*F* (1,507) = 0.090, *p* = 0.764] (see Table 3). Also the total treatment time of patients suffering from schizophrenia did not differ significantly [*F* (1,114) = 0.777, *p* = 0.380]. Mean values are presented in Table 3.

**Table 3:** Total treatment duration, treatment duration in protected ward, treatment duration in open ward, number of stays, circumstances of discharge, admission to day-care clinic before and after the implementation of Soteria-elements.

	**2016**	**2019**	**Statistics**
Total sample size (*N*)	341	173	
Diagnosed with schizophrenia (*n*)	66	51	
**Total treatment duration in days (M** ±**SD)**			
Total sample	20.39 (±20.69)	20.10 (±17.44)	*F* (1,507) = 0.090, *p* = 0.764
Schizophrenia	30.18 (±29.21)	23.25 (±17.91)	*F* (1,114) = 0.777, *p* = 0.380
**Voluntary treatment duration in protected ward in days (M** ±**SD)**			
Total sample	9.77 (±15.15)	1.17 (±3.14)	*F*(1,507) = 56.043, *p* < 0.001^***^
Schizophrenia	14.80 (±28.27)	0.27 (±1.01)	*F*(1,114) = 12.606, *p* < 0.001^***^
**Treatment duration in open ward in days (M** ±**SD)**			
Total sample	10.60 (±18.05)	18.94 (±17.15)	*F*(1,507) = 31.805, *p* < 0.001^***^
Schizophrenia	15.38 (±21.86)	22.98 (±17.92)	*F*(1,114) = 7.532, *p* = 0.007^**^
**Multiple stays per year in % (yes/no)**			
Total sample	37.2/62.8	37.0/63.0	χ^2^(1) = 0.003, *p* = 0.956
Schizophrenia	45.5/54.5	31.4/68.6	χ^2^(1) = 2.391, *p* = 0.122
**Number of stays per year (*****M*** ±**SD)**			
Total sample	1.71 (±1.38)	1.75 (±1.69)	*F* (1,507) = 0.074, *p* = 0.786
Schizophrenia	1.79 (±1.31)	1.63 (±1.67)	*F* (1,114) = 0.346, *p* = 0.557
**Circumstances at discharge in % (by agreement—discontinuation—against medical advice)**			
Total sample	87.4/3.2/9.4	75.7/13.3/11.0	χ^2^(2) = 19.759, *p* < 0.001^***^
Schizophrenia	95.5/1.5/3.0	78.4/13.7/7.8	χ^2^(2) = 8.520, *p* = 0.014^*^
**Admission to day-care clinic in % (yes/no)**			
Total sample	8.8 91.2	13.9/86.1	χ^2^(1) = 2.382, *p* = 0.123
Schizophrenia	15.2/84.8	17.6/82.4	χ^2^(1) = 0.132, *p* = 0.717

*n*, Number of subjects; *M*, mean value; SD, standard deviation;

* *p* < 0.05;

** *p* < 0.01;

*** *p* < 0.001.

#### 3.2.2. Duration of voluntary stay in protected ward

Since 2019 a switch according to the patient's needs between the protected and open area of the ward was possible, the differences between the treatment time in the respective area were analyzed (see Table 3). A global view of all treated patients showed that in 2019 the number of days voluntarily spent in the protected ward was significantly reduced [*F* (1,507) = 56.043, *p* < 0.001].

This effect persisted in individual consideration of patients with schizophrenia [*F* (1,114) = 12.606, *p* < 0.001]

#### 3.2.3. Duration of stay in open area

To explore the effect of Soteria-elements on treatment time in the open area, a variable of total open treatment time was created. Since 2016 there was no possibility to be treated in an open area on the acute ward, therefore some cases were transferred to other open wards. To maintain comparability, the number of days the respective patients spent on other open wards in 2016 were included in the analysis. In 2019, a significant increase of treatment time in the open sector was noticed [*F* (1,507) = 31.805, *p* < 0.001] over all patients, regardless of diagnosis.

Subgroup analysis showed consistent results for patients with schizophrenia [*F* (1,114) = 7.532, *p* = 0.007] (see Table 3).

#### 3.2.4. Medication dosage at discharge (CPZE)

**Figure 1:**
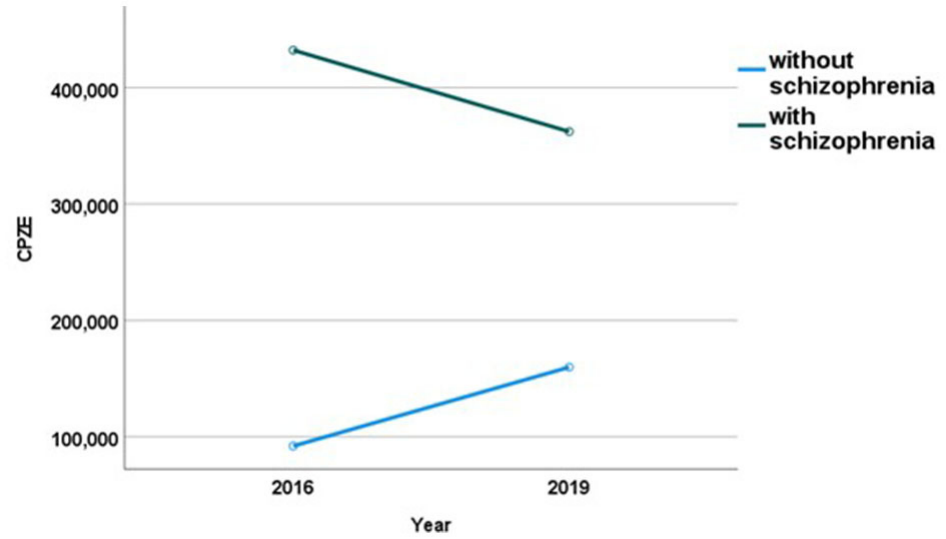
Mean medication dosage in chlorpromazine equivalents (CPZE) at discharge before (2016) and after (2019) the implementation of Soteria-elements.

Comparing the dosage of medication at discharge, CPZE values of antipsychotic discharge medication were generated. Differences in mean CPZE values are presented in Table 4. A univariate two-way ANOVA showed no significant main effect of the year of treatment [*F* (1,509) = 0.263, *p* = 0.609]. The main effect for the existence of schizophrenia on medication dosage is statistically significant [*F* (1,509) = 94.915, *p* < 0.001]. There is a significant interaction between the year and type of diagnosis [*F* (1,509) = 6.358, *p* = 0.012] (see Figure 1). The reduction of medication between the years is significantly moderated by the type of diagnosis, resulting in less medication for patients diagnosed with schizophrenia. The increase of medication for the other groups of patients will be discussed subsequently.

**Table 4:** Medication dosage at discharge in chlorpromazine equivalents (CPZE) (M ± SD) before and after the implementation of Soteria-elements.

	**CPZE (*****M*** ±**SD)**		**Statistics**
**Main effects**			
**Year of treatment**			
2016	157.91 (±279.74)		*F* (1,509) = 0.263, *p* = 0.609
2019	219.46 (±294.94)		
**Type of diagnosis**			
Without schizophrenia	112.90 (±232.21)		*F* (1,509) = 94.915, *p* < 0.001^***^
With schizophrenia	401.68 (±336.25)		
**Interaction**	2016	2019	
Without schizophrenia 2016 *n* = 275 2019 *n* = 122	92.09 (±211.14)	159.82 (±268.91)	*F* (1,509) = 6.358, *p* = 0.012^*^
With schizophrenia 2016 *n* = 66 2019 *n* = 51	432.18 (±355.94)	362.21 (±308.87)	

*n*, Number of subjects; M, mean value; SD, standard deviation; ^*^p < 0.05; ^***^p < 0.001.

#### 3.2.5. “Revolving door effect” and number of stays per year

Comparing all patient groups, there was neither a significant difference between the number of patients with multiple stays per year (“revolving door effect”) [χ^2^(1) = 0.003, *p* = 0.956], nor a significant change in the number of admissions per patient in 2016 or 2019, respectively [*F*(1,507) = 0.074, *p* = 0.786].

Concurrently, neither did the number of patients diagnosed with schizophrenia with multiple stays per year diminish significantly in 2019 [χ^2^(1) = 2.291, *p* = 0.122], nor did the number of stays per year per patient of this group differ significantly between the years [*F*(1,114) = 0.346, *p* = 0.557] (see Table 3).

#### 3.2.6. Circumstances of discharge

To compare the circumstances in which the patients ended the inpatient treatment, a variable with three categories was created: (1) discharge by agreement (including planned discharges, discharges upon the patient's own request, transfer to further external treatment), (2) discontinuation (premature termination, no further treatment offer), and (3) against medical advice. Relative frequencies are shown in Table 3. Results show a significant difference between the years for all patients [χ^2^(2) = 19.759, *p* < 0.001], resulting in an increase of premature termination in 2019.

Patients diagnosed with schizophrenia ended the treatment significantly more often prematurely [χ^2^(2) = 8.520, *p* = 0.014], (compare Table 3).

#### 3.2.7. Admission to day-care clinic

Direct admission to the hospital's day-care clinic after inpatient treatment was assessed (relative frequencies are reported in Table 3). Over all treated patients, the admission rate between the years did not change significantly [χ^2^(1) = 2.382, *p* = 0.123].

The difference of patients with schizophrenia admitted to day-care clinic between the years is not statistically significant [χ^2^(1) = 0.132, *p* = 0.717] (see Table 3).

## 4. Discussion

Results suggest that inpatient treatment with Soteria-elements is not only feasible but also beneficial in terms of a less restricted and harmful treatment experience in an acute psychiatric ward. The subject of evaluation is the only acute psychiatric ward in the county Oberhavel where Soteria-elements were implemented in 2017. This means that selecting patients was not possible—all acutely ill patients in need of treatment had to be admitted.

A more homogenous population of patients was necessary in order to enable us to offer a more psychosis specific treatment on the acute ward. After the reconstruction, the distribution of diagnoses was thus significantly different. In 2019, an increase in the number of patients with schizophrenia spectrum disorder and acute psychotic disorder was notable. When added to the legally accommodated patients [treatment outcomes are presented in a previous article (10)], this group forms the majority of patients treated on the acute ward. This is in keeping with the requirements of the Soteria Fidelity Scale (12) for a ward with Soteria-elements.

Since the distribution of diagnoses changed significantly between the years, comparisons of all other patients must be interpreted with care. Still, the presented data offer insights into how a less restricted, more recovery-oriented treatment is possible for all patients. With respect to the total number of patients, when comparing treatment outcomes before and after the implementation of Soteria-elements, the total treatment duration did not change significantly. However, by creating an alternative ward environment-spatially and therapeutically—all patients were able to spend significantly less days in the protected area and significantly more days in the open area of the ward, regardless of their diagnosis. Comparing the treatment offered in 2016 with that provided in 2019 for all patients suffering from schizophrenia spectrum disorder, the results show that it was possible to reduce the time spent in the protected area and increase the time in the open area. The mean treatment duration of 23 days in 2019 of this subgroup was much shorter when compared to other Soteria projects, who report 38 to 63 days (20, 21) of total treatment time.

We can only speculate as to why there was a significant decrease of mean age in the whole patient group. This applies to the whole group of patients as well as to the schizophrenic patients. In all calculations, age was integrated as a covariate.

Patients with diagnosed schizophrenia spectrum disorder appear to benefit more from the setting than other patient groups with respect to medication dosage. Lower medication dosages were significantly linked to the group of diagnosis, favoring schizophrenia. A reduction of medication for patients with schizophrenia is in line with the demands of Mosher and Ciompi, who were able to show decreasing medication dosages in Soteria housing (1, 6). Also, the fact that the reduction of medication dosage is notable for this particular patient group, but not for patients suffering from different diagnoses supports Ciompi's hypothesis that Soteria-treatment might specifically have a stress reducing effect for psychotic patients (3, 4), subsequently allowing lower medication dosages. In Hennigsdorf hospital, patients suffering from schizophrenia were discharged in 2019 with approximately 70 CPZE less than in 2016. This corresponds approximately to 1.5 mg risperidone or 50 mg quetiapine per day. The medication dosage in the Hennigsdorf hospital is comparable to or even below the mean dosage of 450 CPZE for acutely ill psychiatric patients in a Norwegian health study (22). Furthermore, our results show a slight increase of medication dosage for patients with diagnoses other than schizophrenia. This might be due to the specialization of the therapeutic concepts of the acute ward and the other psychiatric wards. We tended to admit patients primarily to the respective specialized ward. Thus, we assume that those who were still admitted in 2019 to the acute ward needed more intense and high frequent treatment corresponding to group Psy_2_ in the platform model (13). This might explain the increase of higher antipsychotic medication dosages for those patients.

Premature discontinuation of inpatient treatment increased significantly in 2019 after reconstruction and implementation of the open doors policy. This applies to the whole group of patients as well as to the subgroup of the patients suffering from schizophrenia spectrum disorder. Research shows inconsistent results regarding the effect of open-door policies on this matter (23). Steinert et al. could observe that in some studies, a reduction of premature discontinuation was notable during open doors, in others early discharges were increasing, or could only be prevented by closing the ward's doors. Since the Hennigsdorf hospital is responsible for the whole catchment area Oberhavel in Brandenburg, monitoring of re-admission rates after early treatment drop-out is easily done. Acutely ill patients admitted to a different hospital in the area will be re-transferred to the responsible hospital promptly. Bearing that in mind, although not statistically significant, the “revolving door effect” has been diminished for the patients with schizophrenia. These findings allow to draw the conclusion that treatment with Soteria-elements including the established relapse prevention might contribute to the success of sustainable treatment of acutely ill patients suffering from schizophrenia. With respect to a planned discharge management, further outpatient treatment options in the county are presented and established right from the beginning of the treatment. This can also be used by patients who leave the inpatient treatment prematurely. These findings are in line with research regarding planned early discharge to prevent long-time hospitalization without increasing the “revolving door effect” (24). It can be assumed that by providing a Soteria-specific day-care clinic in Hennigsdorf hospital in the future, the admission rate of patients with schizophrenia to disorder specific treatment can be further improved. First experiences were made in Bern/Switzerland and the kbo-Isar-Amper-Klinikum Munich/Germany.

In conclusion, treatment with Soteria-elements seems to have a favorable effect on the treatment outcome of psychotic patients (e.g., shorter treatment duration in a locked ward, lower medication dosage) and can thus be evaluated as applicable in a ward where acutely ill patients—repeatedly or recently ill are admitted to. Also, the World Health Organization (WHO) recommended Soteria-treatment as a good clinical practice to foster patients' rights and recovery (25). This corresponds to the claims of the ratification of the UN Convention on the Rights of Persons with Disabilities (26), which demands a more critical application of coercive measures in acute psychiatry. Additionally, a safe and supporting environment as well as transparency and participation during the treatment comply with aspects patients wish for in a crisis (27).

## 5. Limitations

The goal of the study was to evaluate whether Soteria-elements in acute psychiatry made any change to the treatment outcome. Previous authors referring to Soteria always emphasized the assumed beneficial value of Soteria treatment specifically in schizophrenia. However, they had no comparison group. We tried to compare in a pre-post design the actual differences in treatment outcomes before and after the changes on the ward. There are evident limitations due to the fact that the data are based on a retrospective analysis. Thus, a randomization was not possible in this design. Also, the shift of the distribution of diagnoses between the years allows only limited conclusions for the group of all patients. While some comparisons did not reach a level of statistical significance, a trend of changing mean values in favor of Soteria-treatment was notable. This replicates findings from earlier Soteria evaluation studies, which argue that Soteria-treatment appears to be at least equally effective as treatment as usual, while at the same time reducing medication dosages (8). To optimize the future research process regarding Soteria-treatment offers, accompanying research should be carried out before, during, and after the implementation with additional standard measures as the Positive and Negative Syndrome Scale [PANSS (28)] or Global Assessment of Functioning (GAF). Follow-up data would also be helpful to assess the sustainability of the treatment with Soteria-elements compared to the treatment as usual. We understand that there is a large overlap in the guidelines for Soteria treatment and the national guidelines for the treatment in acute psychiatric wards [also see Steinert and Hirsch (29)]. There are several programs in modern psychiatry which concentrate on the prevention of coercion and violence and the increase in participation. In our opinion, Soteria treatment is one approach to comply with those guidelines.

## Data availability statement

The data analyzed in this study is subject to the following licenses/restrictions: The pseudonymised data is retrieved from the hospital's internal documentation system and will be provided upon request. Requests to access these datasets should be directed to philine.fabel@oberhavel-kliniken.de.

## Ethics statement

Ethical review and approval was not required for the study on human participants in accordance with the local legislation and institutional requirements. Written informed consent for participation was not required for this study in accordance with the national legislation and the institutional requirements.

## Author contributions

PF holds first authorship of this article, organized the database, performed the statistical analysis, and wrote the manuscript. TW, HZ, and MJ-S contributed substantially to conception and design of the study. TW and JR supported regarding the statistical analysis. MJ-S holds senior authorship and initiated and supervised the whole project. All authors contributed to manuscript revision, read, and approved the submitted version.

## Footnotes

^1^A sensitivity analysis resulted in four patients, who would not have been included in the data set, if the *post-hoc* data collection strategy would have been used for all patients. Since an exclusion of those four patients caused no differences in the results, *post-hoc* data collection strategy was considered to be reliable and thus permissible. The reported results include the four identified patients who were admitted in an intoxicated state, thus the main diagnose at time of admission was none of the abovementioned categories.
